# Preparation and Characterization of UV-LED Curable Acrylic Films Containing Biochar and/or Multiwalled Carbon Nanotubes: Effect of the Filler Loading on the Rheological, Thermal and Optical Properties

**DOI:** 10.3390/polym12040796

**Published:** 2020-04-02

**Authors:** Valentina Strongone, Mattia Bartoli, Pravin Jagdale, Rossella Arrigo, Alberto Tagliaferro, Giulio Malucelli

**Affiliations:** 1 Department of Applied Science and Technology, and local INSTM Unit., Viale Teresa Michel 5, 15121 Alessandria, Italy; valentina.strongone@polito.it (V.S.); rossella.arrigo@polito.it (R.A.); 2 Department of Applied Science and Technology, C.so Duca degli Abruzzi 24, 10129 Torino, Italy; mattia.bartoli@polito.it (M.B.); alberto.tagliaferro@polito.it (A.T.); 3 Italian Institute of Technology, Via Livorno 60, 10144 Torino, Italy; pravin.jagdale@iit.it

**Keywords:** UV-LED curing, epoxy-acrylate resin, biochar, multiwalled carbon nanotubes, composites

## Abstract

UV-LED curable coatings represent an up-to-date attractive field due to the high curing efficiency even in the presence of high filler loadings, as well as to the absence of infrared wavelengths that may negatively impact on heat-sensitive substrates. The addition of carbonaceous materials, such as biochar (BC) and/or multiwalled carbon nanotubes (MWCNTs) could positively improve both the rheological and thermal properties. In this study we report on the synthesis and characterization of carbon-reinforced films containing nanometric (MWCNTs) and micrometric (BC) carbon-based materials. We analyze the rheological properties of the UV-LED curable dispersions, as well as the thermal and optical properties of the resulting films, establishing some correlations between filler dispersion/loading with the main observed properties.

## 1. Introduction

Photoinduced polymerization processes are very well-established curing methods that exploit high energy radiations for transforming a liquid system into a solid 3D network. This occurs in a very short time (i.e., a few seconds are enough for completing the curing process), with a low energy consumption (necessary just for triggering the curing reaction; unlike thermally-induced processes, there is no need to heat the bulk of the curable system and the process is carried out at room temperature) and without using solvents (as the recipes of photocurable systems usually already comprise reactive diluents that are able to adjust the viscosity while taking part to the curing reactions) [1,2,3,4,5,6,7]. As a result, photocuring processes have found several uses for different industrial applications, comprising varnishes, printing inks and protective coatings on a variety of substrates, including paper, wood, metals, plastics and fabrics. Besides, several high-tech and electronic applications have been thoroughly investigated and also developed at an industrial scale: they include the use of coatings on optical fibers, wave guides and optical recording media, and the fabrication of printed circuit boards [8,9].

UV radiation sources were the first to be designed and fabricated; they are still being employed for several advanced applications, mainly in the field of functional coatings and inks. However, they show some limitations concerning the low efficiency in photocurable pigmented systems or UV-curable composites containing high loadings of filler or reinforcing agent. In fact, all these additives, if the UV-curable recipe is not well tailored and suitable mixtures of photoinitiators are employed, can absorb most of the incident radiation on the sample surface, hence lowering the quantum efficiency and leading to incomplete curing.

This drawback can be overcome by using UV-LED curing systems: unlike UV (i.e., mercury arc) lamps, UV-LED units deliver a monochromatic UV radiation (its wavelength is usually approximately about 400 nm or slightly shorter), displaying almost Gaussian distribution with a relatively narrow bandwidth. Besides, they are mercury-free and they do not generate ozone and therefore they determine a very low environmental impact. Furthermore, the “cold” radiation provided by UV-LED curing systems, mostly due to the absence of output in the infrared region, can be successfully exploited for applications on heat-sensitive substrates (such as wood and fabrics, among a few to mention) [10]. Last but not least, the electrical-to-optical conversion efficiency of UV-LED units is much higher with respect to standard UV lamps and the former can be switched off instantly, hence saving from around 50–75% of electrical power.

To the best of the authors’ knowledge, the number of scientific articles dealing with the use of UV-LED curing processes for the preparation of thermosetting composite systems is at present very limited. Zhang and coworkers studied the effects of the UV-LED exposure dose on the cure homogeneity and interlaminar shear strength of composite laminates derived from prepregs made of E-glass fibers and a mixture of acrylic monomers and oligomers [11].

In another quite recent paper, Faes et al. [12] designed a novel 3D printing method that combines syringe extrusion and UV-LED curing: this way it was possible to produce high density ceramic components, after firing and sintering 3D printed UV-LED curable acrylic systems containing zirconia at different loadings (from 22.5 to 55 vol%).

In a recent work, it was possible to prepare UV-LED curable epoxy-acrylate nanocoatings containing intercalated phyllosilicates with enhanced thermal and thermomechanical features, as well as improved barrier properties towards oxygen diffusion [13].

As the incorporation of micro-to-nano-fillers in photocurable systems has clearly demonstrated its suitability for designing novel materials with improved mechanical, thermal, electrical and barrier properties with respect to the unfilled counterparts, in this work we investigated the effects of the presence of two selected carbon materials, namely multiwalled carbon nanotubes (MWCNTs) and biochar, on the rheological, thermal and optical properties of epoxy-acrylate UV-LED curable systems. Indeed, the scientific literature clearly highlights the exploitability of 1-3D carbonaceous fillers in various fields, as they can approve the properties of a plethora of nanocomposites [14,15,16,17].

The choice of these two carbon-based materials was driven by two main reasons: the first is that biochar is currently emerging as a cheap, functional material already useful for several applications (i.e., as storage for volatile nutrients, as an adsorber in functional clothing, as an insulating material in the building industry, as energy storage in batteries, among a few to mention) and represents a way for recovering wastes at the end of life, providing them with a new added-value [18]. The second reason refers to the shuttle effect that biochar, according to its structure and morphology, may exert on MWCNTs, favoring the dispersion of the latter into a polymer system.

In this work, each carbon-based filler was dispersed in bisphenol-A-ethoxylate-diacrylate (i.e., a commercially available resin, here used as the model system) at different loadings (ranging from 0.01 to 1.0 wt%), by means of a tip sonicator; further, the two additives were also premixed keeping 1:1 weight ratio and added to the resin, aiming at assessing the effect of their concurrent presence on the overall performances of the resulting UV-LED curable systems.

In particular, it was possible to establish some correlations between the rheological, thermal, optical properties of the composite systems and the structure and level of dispersions of the fillers used.

## 2. Materials and Methods

### 2.1. Materials

Exhausted coffee powder was selected as a real case study. It was collected from Bar Katia (Turin, Italy) supplied by Vergnano Spa (Torino, Italy) and corresponds to an Arabica mixture. The exhausted coffee was collected and dried at 105 °C for 72 h. Coffee samples (100 g) were pyrolyzed using a vertical furnace and a quartz reactor (heating rate: 15 °C/min) and kept at 800 °C for 30 min in argon atmosphere [19,20,21].

A commercially available epoxy-acrylate resin, Ebecryl 150 (bisphenol-A-ethoxylate-diacrylate hereinafter coded as EB150) was kindly supplied by Allnex (Brussels, Belgium). 2,4,6-Trimethylbenzoyl-diphenylphosphineoxide, herein after coded as TPO, was kindly supplied by IGM Resins (Mortara, Italy) and used as photoinitiator for the UV-LED curing.

MWCNTs (NC7000^TM^, produced via the Catalytic Chemical Vapor Deposition (CCVD) process) were purchased from Nanocyl SA (Sambreville, Belgium). Carbon purity: 90%; surface area: 250–300 m^2^/g, as indicated in the datasheet provided by the supplier.

### 2.2. Preparation of the UV-LED Curable Mixtures

Biochar was grinded using a mechanical mixer (Savatec BB90E) for 10 min at 300 rpm at room temperature in order to decrease the particle size according to Giorcelli et al. [20].

The carbonaceous materials (MWCNTs, Biochar) were dispersed in EB150 with final concentrations of 0.01, 0.10, 0.50 and 1.0 wt%, using a tip Sonics Vibra-cell ultrasonicator (Sonics and Materials, Inc., Newtown, CT, USA) for 15 min. In order to avoid the instantaneous temperature rise, ultrasounds were pulsed with cycles of 10 s alternating to 10 s pause to favor a better heat diffusion. Another set of samples was prepared mixing MWCNTs and biochar (1:1 weight ratio) using the same filler loadings employed for each single carbon material. Then, 6 wt% of TPO was added to the UV-LED curable mixture.

The stability of the resulting liquid dispersions was monitored by visual inspection up to 4 days after the ultrasonication process: no sedimentation of the fillers was observed during this time. Then, the dispersions were coated on glass plates using a wire-wound applicator: the thickness of the obtained coatings was about 150 μm. The coated glass plates were then subjected to the UV-LED curing process, using a Heraeus Noblelight UV-LED NC1 unit (Cambridge, UK), working in dynamic conditions (belt speed: 1 m/min), at 395 nm, with radiation intensity on the sample surface of about 4.8 W/cm^2^.

### 2.3. Characterization Techniques

The completeness of the UV-LED curing reaction was assessed by means of a Perkin Elmer Spectrum 100 spectrometer (Shelton, Connecticut, USA) equipped with an attenuated total reflection (ATR) diamond probe. FTIR spectra were recorded at wavelengths from 700 to 4000 cm^−1^, with 4 cm^−1^ resolution; 16 scans were collected.

The morphology of all the samples was investigated using a FESEM—Field Emission Scanning Electrical Microscope Zeiss Supra-40 (Carl Zeiss IMT, Oberkochen, Germany).

Rheological measurements were performed using an ARES (TA Instrument, New Castle, DE, USA) strain-controlled rheometer in parallel plate geometry (plate diameter: 25 mm). Strain sweep tests were carried out at 30 °C and ω = 1 rad/s. The complex viscosity and storage and loss moduli were measured performing frequency scans from 10^−1^ to 10^2^ rad/s at 30 °C. The strain amplitude was selected for each sample in order to fall in the linear viscoelastic region. At least two specimens for each system were tested and the results were averaged (standard deviation < 5%).

Differential scanning calorimetry (DSC) analyses were performed using a QA1000 TA Instrument apparatus (TA Instrument Inc., Waters LLC, New Castle, DE, USA). All the experiments were performed under dry N_2_ gas (flow: 50 mL/min) using samples of about 10 mg in sealed aluminum pans. All the films underwent the following cycle: (1) heating from 0 to 160 °C at 10 °C/min; (2) cooling down to 0 °C at 10 °C/min and (3) heating from 0 to 160 °C at 10 °C/min. The glass transition temperature (*T*_g_) was measured as the midpoint of heat capacity changes. Calibration was performed using indium as standard (*T*_m_ = 156.4 °C; Δ*H*_m_ = 28.15 J/g).

Thermogravimetric analyses (TGA) were performed using a Pyris1TGA apparatus (Perkin Elmer, Waltham, MA, USA; experimental error: ±0.5 wt%, ±1 °C). Samples (about 10 mg) were placed in alumina pans and runs were carried out in the range 50–700 °C, with a heating rate of 10 °C/min, under both N_2_ and air flow (35 and 25 mL min^−1^, respectively). *T*_5%_, *T*_10%_ (i.e., the temperatures, at which 5% or 10% weight loss, respectively, occurs) and *T*_max_ values were calculated; besides, the final residue at 700 °C was measured.

Thermal conductivity measurements were carried out with TPS 2500S apparatus (Hot Disk AB, Göteborg, Sweden), equipped with a Kapton sensor (radius 3.189 mm), using the transient plane source (TPS) method [22]. The test temperature (23.00 ± 0.01 °C) was controlled by a silicon oil bath (Haake A40, Thermo Scientific Inc., Austin, TX, USA) equipped with a temperature controller (Haake AC200, Thermo Scientific Inc., Austin, TX, USA).

UV-Vis spectroscopy measurements were performed on the UV-LED cured films by using a Shimadzu UV-Vis spectrophotometer UV2600 series (Shimadzu Italia Srl, Milano, Italy); wavelength range was set between 200 and 1200 nm.

## 3. Results and Discussion

**Figure 1:**
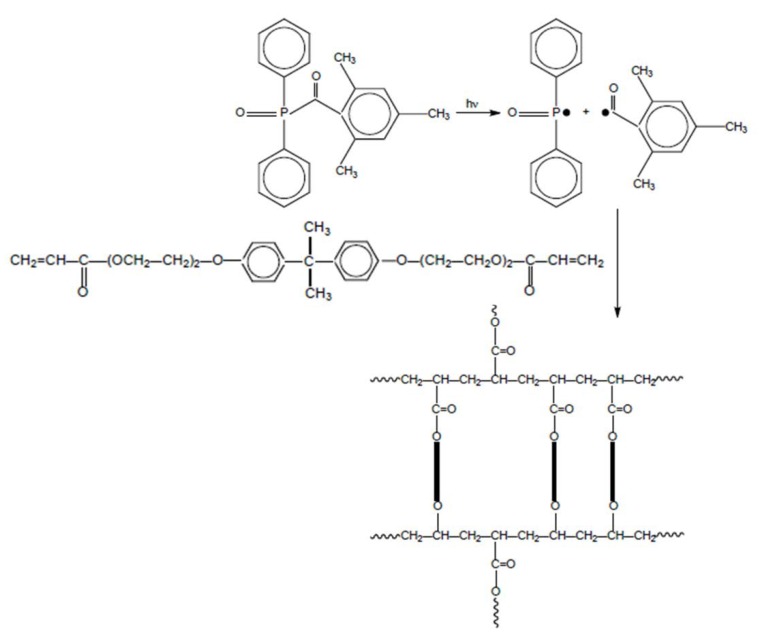
Scheme of UV-LED curing of EB150. The system undergoes a radical polymerization promoted by the generation of primary radicals derived from the photolysis of the photoinitiator as a consequence of the exposure to the UV-LED radiation.

As first point of discussion, a schematic overview on the radical polymerization mechanism of EB150 is shown in Figure 1.

**Figure 2:**
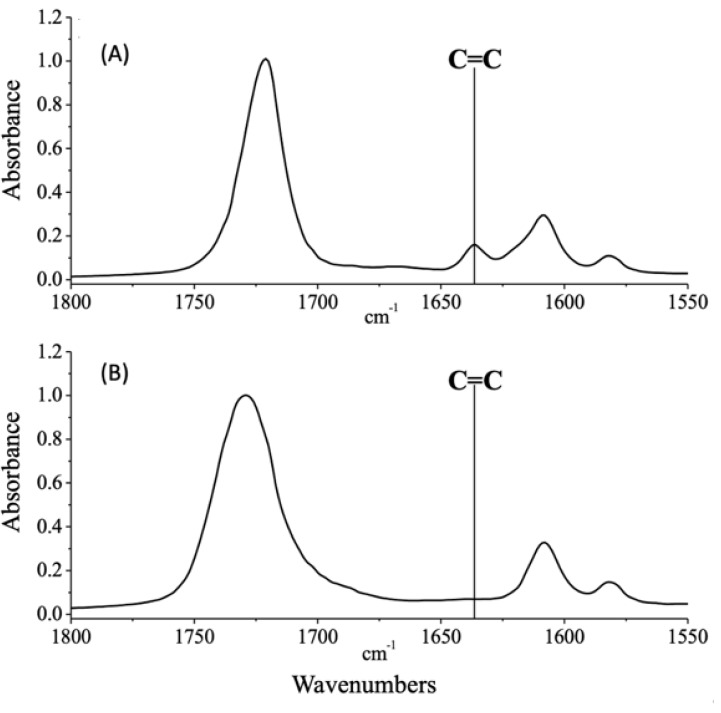
FTIR-ATR spectra of EB150 containing 0.50 wt% of biochar, before (**A**) and after (**B**) exposure to the UV-LED radiation. The disappearance of the band of the acrylic double bonds, located at about 1635 cm^−1^, is a clear indication of the completeness of the UV-LED curing process.

The curing kinetics is very fast, due to the high efficiency of TPO photoinitiator and the high intensity of the UV-LED unit, whose emission spectrum well matches the absorption spectrum of TPO; besides, the adopted experimental conditions for the photocuring process were suitable for achieving the completeness of the double bonds conversion, as revealed by FTIR-ATR spectra before and after the exposure to the UV-LED radiation. As an example, the typical spectra for the system containing 0.50 wt% of biochar, before and after UV-LED curing are shown in Figure 2: the complete disappearance of the band at 1630 cm^−1^ (attributed to the acrylic double bonds [23]) is evident.

**Figure 3:**
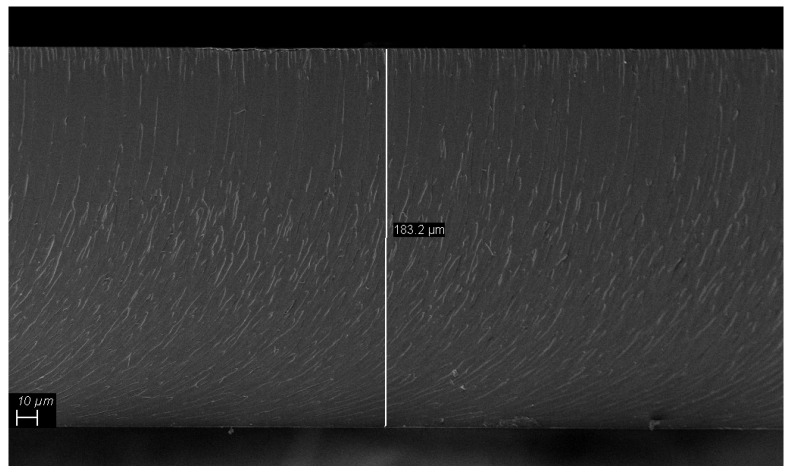
FESEM capture of unfilled UV-LED cured EB150 film on the transversal plane.

The thickness of the obtained films estimated though FESEM technique, irrespective of their composition and filler loadings, was about 180 μm as shown in Figure 3.

### 3.1. Morphological Characterization of the Fillers

**Figure 4:**
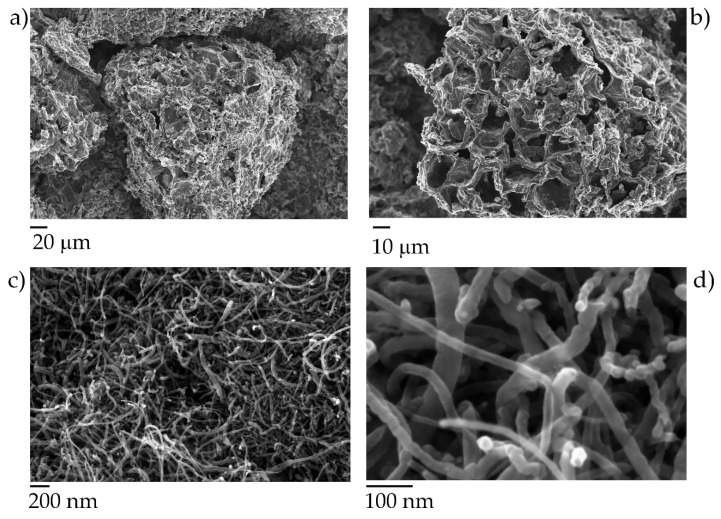
FESEM captures of BC (**a**,**b**) and multiwalled carbon nanotubes (MWCNTs; **c**,**d**) at different magnifications. It is worthy to note the highly porous structure of Biochar

The carbonaceous fillers used were very different in both size and morphology, as clearly shown in FESEM images presented in Figure 4.

**Figure 5:**
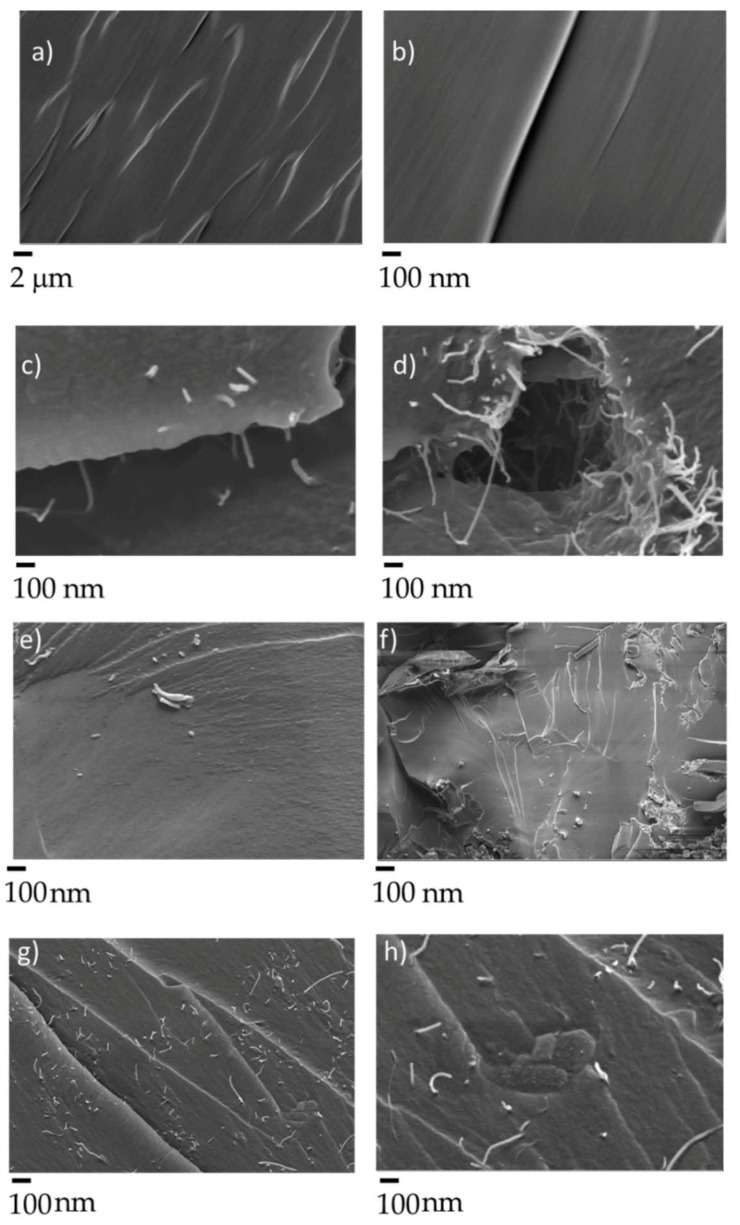
FESEM captures of UV-LED cured films (filler loading: 1 wt%): unfilled EB150 (**a**,**b**); EB150 + MWCNTs (**c**,**d**); EB150 + biochar (**e**,**f**) and EB150 + biochar/MWCNTs (**g**,**h**). All the filled UV-LED cured films show a good dispersion of the two carbonaceous fillers.

BC was produced by carbonization of exhausted coffee following the procedure previously reported [14]. The resulting material showed a sponge-like porous structure with a grain size around 200–300 μm, average radius about 10–15 μm and a wall thick between 1 and 2 μm. According to Giorcelli et al. [20], the structure of BC is quite disordered and does not show residual functional groups (i.e., hydroxyl, quinoid and carboxyl functionalities). The employed MWCNTs are highly packed with an average diameter highly variable in the range from 10 to 50 nm with a length of several micrometers. This network structure deeply affects their dispersibility in organic matrix. Nonetheless, ultrasonic procedures can be effectively exploited to achieve good dispersion within the epoxy-acrylate resin [24]. Besides, using pulsed ultrasounds generated by the tip sonicator allowed achieving good dispersion without compromising the size of MWCNTs. On the other hand, ultrasonication always leads to a sized reduction of biochar, as proved by Bartoli et al. [25], who tested several biochars with different shape and size. However, an acceptable dispersion in EB150 was achieved for both the fillers and their mixtures as shown in Figure 5.

The unfilled UV-LED cured resin shows very smooth surfaces without the presence of any appreciable defects. The addition of MWCNTs at 1.0 wt% (Figure 5c–d) revealed some persistent aggregation but a good embedding of the nanofiller into the polymer matrix. The biochar-containing samples (Figure 5e–f) show a very good dispersion of the particles that, during sonication, underwent a disruptive process, showing surfaces very similar to the neat resin. Interestingly, when BC was mixed with MWCNTs, an improvement of MWCNTs dispersion was achieved as clearly presented in Figure 5g–h. This could be reasonably due to the shuttle effect of micrometric particles of biochar. In particular, in the very first stage of the mixing process, MWCNTs strongly interact with BC surface through π–π stacking: a similar behavior was observed by Ismail et al. [26] using a mix of CNTs and carbon black. In a subsequent step, BC particle are reduced in size due to the sonication process and the surface interacting MWCNTs are homogeneously dispersed within the resin. This beneficial effect could explain the increased uniformity of filler dispersion, accompanied by the decrease of MWCNTs length.

### 3.2. Rheological Behavior

**Figure 6:**
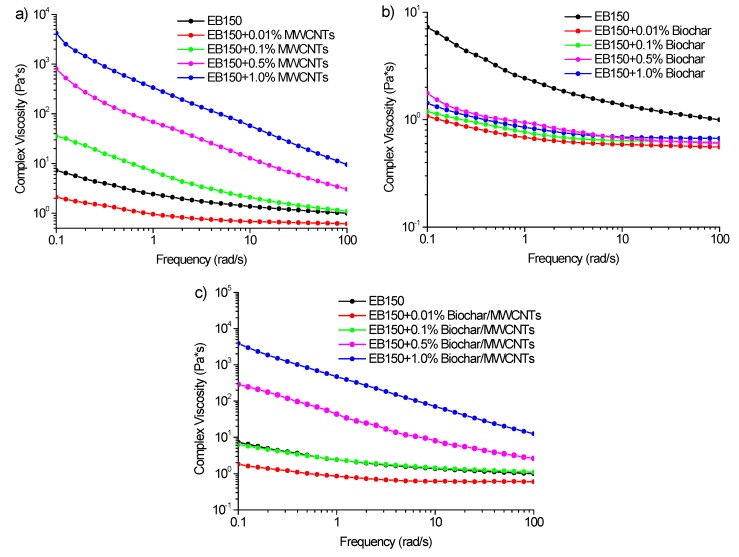
Complex viscosity vs. frequency curves for unfilled EB150 and EB150 + MWCNTs (**a**), EB150 + biochar (**b**) and EB150 + biochar/MWCNTs (**c**) at different loadings ranging from 0.01 to 1.00 wt%.

Figure 6a–c shows the trends of complex viscosity as a function of frequency for EB150 and its mixtures with biochar, MWCNTs and biochar/MWCNTs at different filler loadings. First of all, it is worthy to note that the systems loaded with MWCNTs exhibit a high increase of complex viscosity compared with unfilled resin; the only exception is for the liquid dispersion containing 0.01 wt% of MWCNTs. This finding can be ascribed to the highly entangled structure taking place between π orbital system of MWCNTs and bisphenol A rings, hence to increasing interactions occurring between the oligomeric chains and the nanofiller. Conversely, 0.01 wt% of MWCNTs is too low for ensuring the occurrence of these interactions and the related complex viscosity curve is shifted downwards.

Conversely, for the systems containing biochar at different loadings, the complex viscosity curves shift towards lower values with increasing the filler loading. This finding clearly indicates that biochar exerts a lubricating action on the oligomeric chains, which is more pronounced as the filler loading increases.

Further, a balance in between these two opposite rheological behaviors is shown by the mixtures that combine together biochar and MWCNTs (Figure 6c). In this case, at low loadings (i.e., 0.10 wt%) the complex viscosity trend was mainly driven by the lubricating effect of biochar, as the complex viscosity values were lower or comparable with those of unfilled EB150. At variance, for higher fillers loadings, MWCNTs became prevalent on determining the rheological behavior of the dispersions: in fact, the complex viscosity curves approach those obtained for the systems containing MWCNTs only.

### 3.3. Properties of the UV-LED Cured Films

#### 3.3.1. Thermal Properties

**Figure 7:**
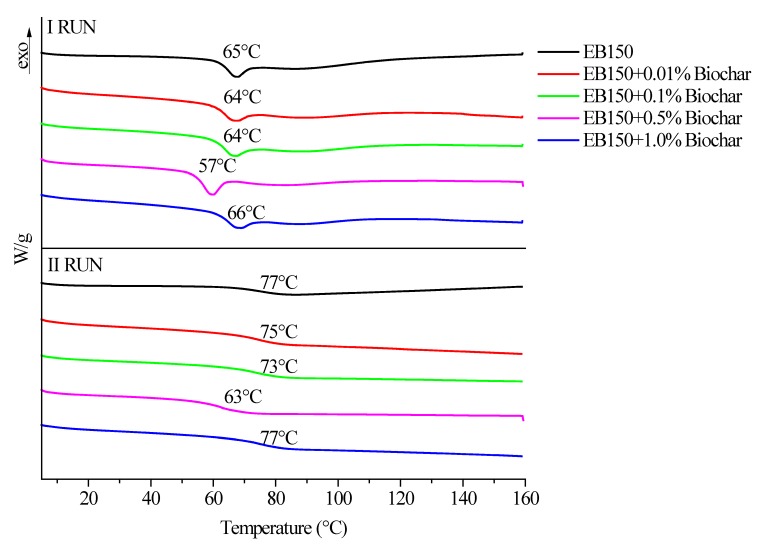
Differential scanning calorimetry (DSC) traces (1st and 2nd heating up) for UV-LED cured EB150 and its composites with different biochar loadings.

**Figure 8:**
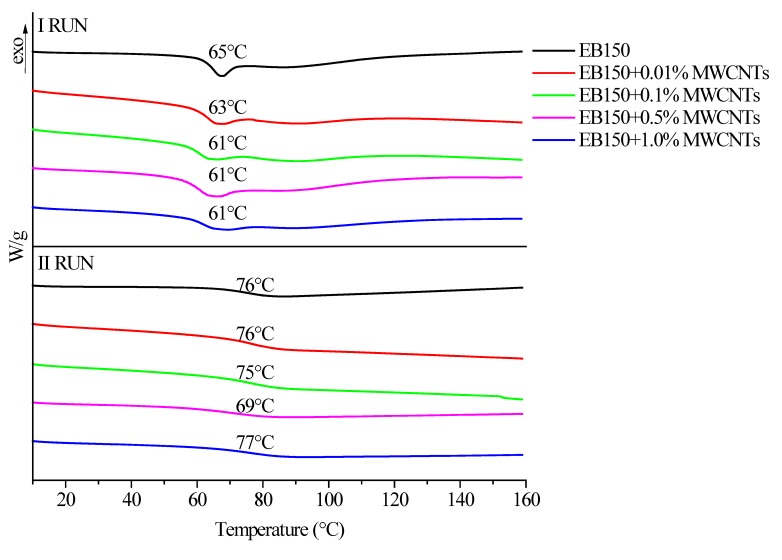
DSC traces (1st and 2nd heating up) for UV-LED cured EB150 and its composites with different MWCNTs loadings.

**Figure 9:**
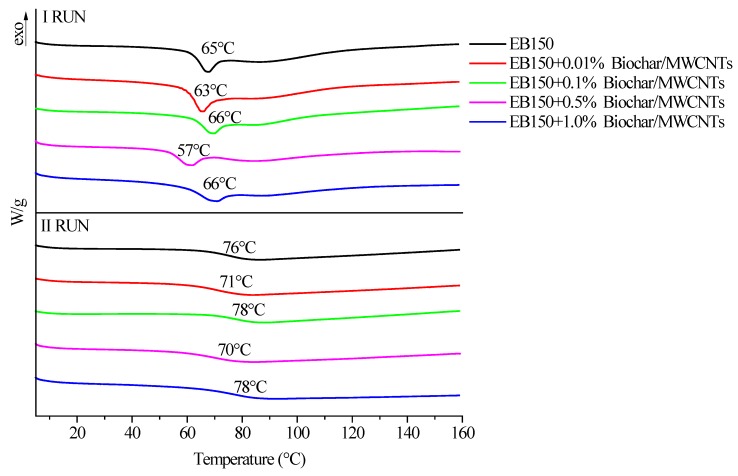
DSC traces (1st and 2nd heating up) for UV-LED cured EB150 and its composites with different MWCNTs loadings.

First of all, DSC analyses were performed on all the prepared systems, in order to (i) confirm the completeness of the UV-LED curing reaction (1st heating up—absence of exothermic peaks above the glass transition temperature) and (ii) measure the glass transition temperature (2nd heating up). Figure 7, Figure 8 and Figure 9 plots the typical DSC traces for UV-LED cured EB150 and for all the prepared composite films, together with the *T*_g_ values measured as the midpoint of heat capacity steps.

First of all, it is worthy to note that no exothermic effects are detectable in any 1st heating up for UV-LED cured EB150 and its composites: this finding further supports the FTIR-ATR results as far as the completeness of the curing reaction is considered. Therefore, it can be concluded that the experimental conditions adopted for the UV-LED curing (i.e., radiation intensity on the films surface and belt speed) are adequate for promoting the formation of a fully cured network, even in the presence of the fillers. Conversely, the first heating up traces show the enthalpy relaxation (i.e., the endothermic peaks shown in Figure 7, Figure 8 and Figure 9—1st run), which superimposes to the specific heat step associated with the glass transition of the polymer network. The presence of this phenomenon is likely to be attributed to the speed of the UV-LED curing process that freezes the macromolecules in a non-equilibrium thermodynamic state. Then, the 2nd heating up allows the free macromolecule segments between cross-links to rearrange in a more stable conformational structure and enthalpy relaxation disappears. This phenomenon has been already observed for similar systems containing different ZnO fillers [27].

From an overall point of view, the presence of the different carbon fillers at different loadings did not significantly affect the *T*_g_ values of the UV-LED cured polymer network, which were usually within 70 and 77 °C.

Then, all the UV-LED cured films were analyzed through thermogravimetric (TG) analysis carried out in nitrogen and air, in order to investigate the effect of the carbon-based fillers on the thermal and thermo-oxidative stability of the UV-LED cured resin, respectively. The collected data are summarized in Table 1.

In nitrogen atmosphere, degradation occurs according to a single step. Comparing the behavior of the unfilled cured resin with that of its composites, the presence of a filler, irrespective of the type and loading, did not affect the thermal stability of the cured polymer network. In fact, the changes of *T*_10%_, *T*_50%_ and *T*_max_ (i.e., the temperatures, at which 10% and 50% weight loss occurs and corresponding to the maximum weight loss rate in derivative—dTG—curves, respectively) in the presence of the different fillers are practically negligible, despite an increase of the residues of the filled systems at the end of the test as compared to UV-LED cured EB150.

In air atmosphere, the degradation occurs according to three successive steps. The first one, taking place at about 375 °C, can be related to the decomposition of low molecular weight structures, such as dimer and trimers present in the polymer network. Then, at higher temperatures (i.e., about 440 °C), the main degradation of the polymer network occurred. Finally, during the last stage (at about 560 °C), the degradation products of the previous step were further oxidized, giving rise to the formation of CO and CO_2_. Comparably to what was already observed in nitrogen, the presence of the different carbonaceous filler did not affect the thermo-oxidative stability of the UV-LED cured networks, apart from an increase of the residues at the end of the tests.

**Figure 10:**
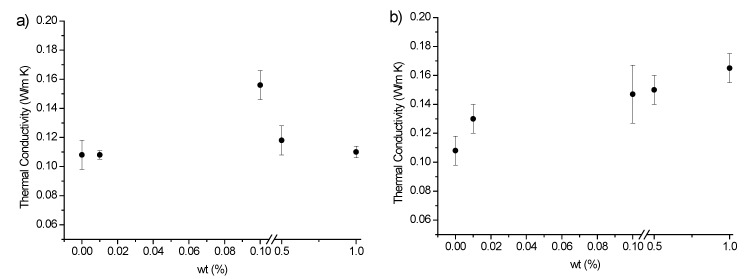

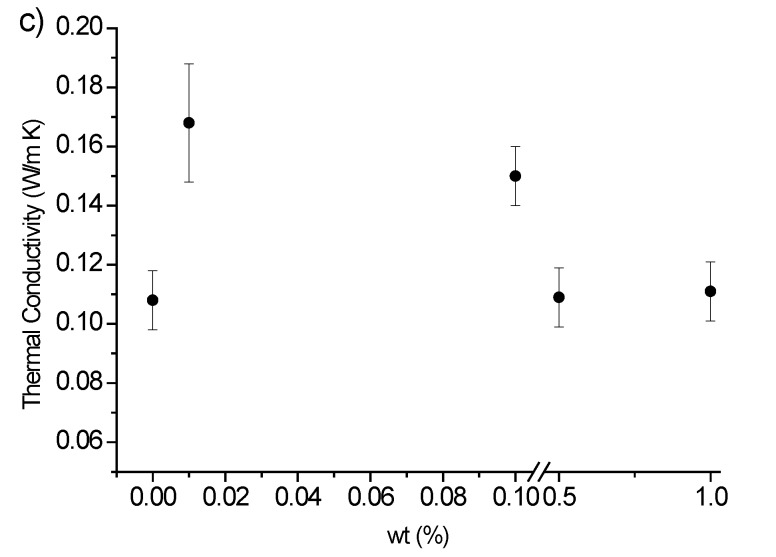
Thermal conductivity of UV-LED cured EB150 and its composite films as a function of filler loading: (**a**) with MWCNTs, (**b**) with biochar and (**c**) with biochar/MWCNTs. Different trends can be observed, according to the filler type and loading.

Figure 10a–c shows the thermal conductivity values as a function of the filler type and loading.

The thermal conductivity data clearly shows a different effect provided by MWCNTs and by biochar. More specifically, the addition of increasing amounts of MWCNTs increased the thermal conductivity that reached a maximum corresponding to 0.10 wt% loading; then, for higher filler loadings, it monotonically decreased. Conversely, the addition of biochar to the UV-curable system determined a monotonically increase of the thermal conductivity, which approached 0.165 Wm^−1^K^−1^ for the highest filler loading. Clustering phenomena occurring with increasing the nanofiller concentration might explain the trend of the thermal conductivity of the films containing MWCNTs: these phenomena have already been observed in FESEM analyses. The latter have also pointed out that biochar shows a very homogeneous dispersion within the polymer network, irrespective of its loading: as a consequence, the thermal conductivity shows a monotonic increasing trend. Finally, the concurrent presence of the two carbon fillers was affected by the different morphology of biochar and MWCNTs in UV-LED cured EB150. In particular, at low fillers loadings, the prevailing effect is exerted by biochar, so that thermal conductivity increases; then, the worse dispersion and clustering tendency of MWCNTs becomes predominant and the thermal conductivity significantly decreases approaching that of unfilled EB150 [28].

#### 3.3.2. Optical Properties

**Figure 11:**
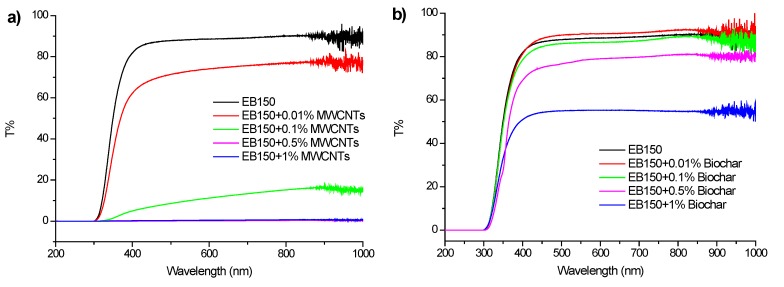

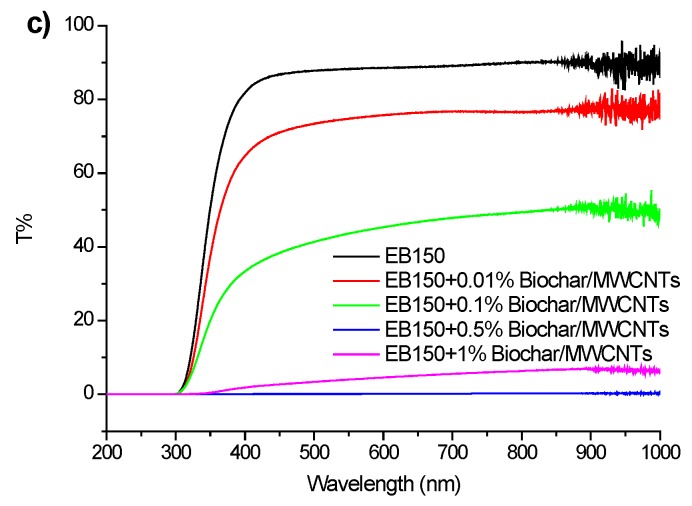
UV-Vis spectra of films containing (**a**) MWCNTs, (**b**) BC and (**c**) BC/MWCNTs in concentrations ranging from 0.01 to 1.00 wt%.

The transmittance over a wide wavelength range of self-standing composite films was evaluated, assessing the effect of the different fillers and of their loadings on the optical properties of UV-LED cured blank counterparts. To this aim, UV-Vis spectroscopy measurements were performed; the obtained data are shown in Figure 11a–c.

From an overall point of view, it is worthy to note that the ease of dispersibility of the fillers within the polymer network significantly affected the transmittance of the resulting composite films, especially when the filler loading was quite high. In particular, unlike MWCNTs, for which the transparency was maintained at very low filler loadings (i.e., 0.01 wt%) only because of clustering phenomena, biochar-containing films were able to keep 50% transmittance at the highest loading (i.e., 1.0 wt%). Finally, the combination of the two fillers did not provide significant enhancements in transmittance, probably due to the light scattering of MWCNTs that limit the beneficial effects provided by biochar.

## 4. Conclusions

In this work, UV-LED curing was exploited for designing new epoxy-acrylate films containing different amounts (ranging from 0.01 to 1.0 wt%) of carbon-based micro and nanofillers, namely biochar and MWCNTs. The UV-LED curing performed in dynamic configuration allowed achieving the completeness of the double bond conversion, even in the presence of the highest fillers’ loadings. The rheological behavior of the UV-LED curable dispersions, as well as the morphology, the thermal and optical properties of the resulting UV-LED cured films were thoroughly investigated and correlated with the type of filler and its loading. In particular, unlike MWCNTs, which beyond 0.01 wt% were prone to clustering, biochar, irrespective of the used concentration, turned out to easily disperse into the UV-LED curable resin, as assessed by FESEM analyses. Besides, the presence of the fillers, regardless of the type and loading, did not affect the thermal and thermo-oxidative stability of the cured films; furthermore, *T*_g_ values were almost unchanged. According to their ease of dispersibility within the polymer network, MWCNTs and biochar showed a different effect on the thermal conductivity of the prepared films: in particular, increasing loadings of biochar providing a monotonic increasing trend of this parameter.

Finally, all the prepared UV-LED films exhibited a high transparency only at low filler loadings; however, the very homogeneous distribution of biochar in the cured resin allowed maintaining high transmittance (about 50%) even at 1.0 wt%, over a wide wavelength range.

Table 2 summarizes all these findings for an easier comparison of the obtained results for the different systems investigated.

## Figures and Tables

**Table 1:** Thermal and thermo-oxidative stability of the UV-LED EB150 film and its composites *.

Filler	Atmosphere: N_2_						Atmosphere: Air					
	Filler Loading (wt%)	*T*_10%_ (°C)	*T*_50%_ (°C)	*T*_max_ (°C)	Residue @*T*_max_ (wt%)	Residue 700 °C (wt%)	*T*_10%_ (°C)	*T*_50%_ (°C)	*T*_1max_ (°C)	*T*_2max_ (°C)	*T*_3max_ (°C)	Residue 700 °C (wt%)
-	0.00	395	439	448	34.6	3.6	349	439	382	438	557	0.3
MWCNT_S_	0.01	393	439	447	35.5	3.6	341	437	382	436	556	0.2
	0.10	394	440	449	33.7	3.7	342	440	383	439	559	0.3
	0.50	392	434	441	37.5	4.1	331	441	378	440	565	0.8
	1.00	395	438	444	39.0	5.0	335	434	379	438	558	0.8
Biochar	0.01	393	438	445	36.5	3.4	346	440	382	437	559	0.3
	0.10	393	438	447	34.1	3.2	342	438	380	438	557	0.4
	0.50	391	437	445	37.4	5.1	350	437	387	437	557	0.4
	1.00	383	432	441	36.2	4.8	340	439	373	439	557	0.7
Biochar/MWCNTs	0.01	394	439	448	34.4	3.6	346	440	382	437	559	0.3
	0.10	391	437	445	36.7	3.5	342	438	380	438	557	0.4
	0.50	390	438	443	40.3	4.8	339	437	377	438	559	0.6
	1.00	388	434	440	38.9	4.7	340	439	373	439	557	0.7

* experimental error: ±0.5 wt%, ±1 °C.

**Table 2:** Main outcomes from the investigated systems.

	BC	MWCNTs	BC + MWCNTs
Completeness of the UV-LED curing process	Achieved	Achieved	Achieved
Dispersibility in EB150	Good	Average; tendency to form aggregates beyond 0.01 wt%	Good; shuttle effect on MWCNTs exerted by BC
Rheological behavior of the liquid dispersions	Lubricating effect at any loading	High increase of complex viscosity beyond 0.01 wt%	Driven by BC at low loadings
Effect of the filler on *T*_g_	Negligible	Negligible	Negligible
Effect on thermal stability	Negligible	Negligible	Negligible
Effect on thermal conductivity	Monotonic increasing trend	Maximum achieved at 0.10 wt% filler loading	Prevailing effect of BC at low filler loadings
Effect on transmittance	50% transmittance kept at 1 wt% loading	High transmittance only below 0.01 wt% loading	Scattering phenomena provided by MWCNTs
